# Exploring the Impact of Individual and Social Antecedents on Teachers’ Teaching Innovation: Perspective of Goal-Oriented Behavior and Social Identity

**DOI:** 10.3389/fpsyg.2022.924052

**Published:** 2022-06-30

**Authors:** Caixia Cao, Beibei Chen, Suping Yang, Xu Zheng, Yan Ye, Xiaoyao Yue

**Affiliations:** ^1^Minnan University of Science and Technology, Quanzhou, China; ^2^East China Normal University Jinan Experimental School, Jinan, China; ^3^School of Education Science, Nanning Normal University, Nanning, China; ^4^Graduate School of Education, Stamford International University, Bangkok, Thailand; ^5^College of Teacher Education, Yuxi Normal University, Yuxi, China

**Keywords:** goal-oriented behavior, teaching innovation intention, innovation performance, social identity, teaching innovation

## Abstract

Many scholars have investigated education management. Scholars in the education field have made significant achievements in contributing to multiple educational reform policies, while other scholars discuss teacher-related issues from the perspective of organizational behavior. The teaching innovation of high school teachers plays a critical role in students’ learning attitude and motivation, especially in the context of the COVID-19 pandemic. Teachers need to utilize more diversified teaching methods to enable students to carry out effective learning. In order to examine teachers’ teaching innovation, this study explores teaching innovation intentions and performance from the perspective of individual and social factors in combination with goal-oriented behavior and social identity theory. This study conducts questionnaires with a sample of Chinese coastal high school teachers, and obtains a total of 475 responses. The research results show that innovation attitude, positive anticipated emotion, group norms and social identity positively affect teachers’ teaching innovation intention; furthermore, teachers’ teaching innovation intention also positively affects their teaching innovation performance. Based on the comprehensive research findings, this research proposes corresponding theoretical and practical implications.

## Introduction

Scholars have widely researched teaching-related topics since teachers’ teaching performance has a huge impact on student learning (Peng and Zhang, 2021; Yang et al., 2021). In particular, teachers’ innovation performance in teaching has been a key topic. Scholars find that the teaching innovation process will create novel and unique learning experiences for students, and helps to flexibly solve learning challenges (Chen et al., 2020; Garzon Artacho et al., 2020). It is important to note that although both learning innovation and learning quality can motivate student learning, there are significant differences. Teaching quality (Fauth et al., 2019) can be achieved through standard processes and routine training (Ohly et al., 2006), but does not necessarily involve creative solutions. However, teaching innovation focuses on how to make students feel “pleasantly surprised” (Mayhew et al., 2021), and highlights how to create new teaching approaches by getting rid of traditional ideas and existing procedures. This study aims to explore the impact mechanism of teachers’ teaching innovation.

This study proposes an explanatory model for service innovation performance by reference to the model of goal-oriented behavior (Perugini and Bagozzi, 2001; Soscia et al., 2018) and social identity theory. A variety of behavioral theories, such as the theory of planned behavior (Ajzen, 1991, 2020; Sahu et al., 2020), believe that individual decisions are mainly affected by three antecedents: innovation attitude, subjective norms and perceived behavior control. Since the theory of planned behavior fails to effectively explain individual decision-making under some circumstances, Perugini and Bagozzi (2001) propose the model of goal-oriented behavior, and further employ it to explain behaviors at the individual level. The model of goal-oriented behavior reveals that the decision-making process of decision makers is more affected by anticipated emotions in addition to the original variables mentioned in the theory of planned behavior. Anticipated emotions are put forward based on the view that humans have “the ability of imagining the future.” In other words, teachers should think students are pleasantly surprised when they deliver innovative teaching; in this case, teachers will appear with positive anticipated emotions, which drives teachers to prepare well for delivering innovative teaching to students. Attitude is a psychological disposition formed based on previous experience or situations (Greenwald and Banaji, 1995), while anticipated emotion is a kind of “psychological emotion” formed when a decision maker anticipates a success. Although relevant behavioral models also consider attitude and anticipated emotions as a concept at the individual’s psychological level, the current service literature often ignores the effect mechanism of this future-focused concept. Many scholars suggest this influencing factor should be considered while discussing the decision-making process of individual behaviors (He et al., 2012; Soscia et al., 2018). This study seeks to deepen previous studies on teaching innovation behaviors through the examination of anticipated emotions. Thus, this study aims to explore the impact of innovative attitude and anticipated emotions on teachers’ teaching innovation intention.

Based on prior studies of innovation performance, this study investigates the impact of social identity on teaching innovation behaviors. Generally speaking, social factors play a role in teaching innovation and delivery plays an advancing role in teaching innovation and delivery (Hughes et al., 2018), because an individual is a part of an organization or team. This argument is in line with views of marketing scholars. For instance, Rather (2018) suggests that social identity has a direct impact on the decision-making process of individual behaviors. Subjective norms can also be regarded as a social factor in the theory of planned behavior; that is, expectations from others will also be considered in the process of making decisions, thus forming a norm-based pressure (Ajzen, 1991, 2020; Kelman, 2006; Sahu et al., 2020). However, the positive social identity provides teachers with a more active driving force. In other words, teachers’ intense identity with schools will further drive teachers to voluntarily put in more efforts for the benefit of schools and for teaching innovation (Chiu et al., 2006). From the academic perspective, the discussion of this antecedent will contribute to studying and comparing the relationships between explanatory variables from different sources and teaching innovation intention. Based on the above arguments, this study aims to explore the impact of group norms and social identity on teachers’ teaching innovation intention.

According to the above explanations, this study proposes relevant research contributions based on the following theoretical gaps: (1) applying goal-oriented behavior and social identity theory to teachers’ teaching, and exploring the effectiveness of teaching innovation; (2) building a conceptual framework to explore antecedents of teachers’ teaching innovation intention from the individual and social perspective, and verify the relevance among them; (3) adopting psychological stimuli to explore the attitude role of teaching innovation intention based on an Asian context.

## Literature Review

### The Model of Goal-Oriented Behavior and Innovation Intention

Goal-oriented behavior focuses on the individual’s control of capabilities and task development, is committed to the intrinsic interest of tasks (Dweck, 1999), and motivates individuals to make sustained efforts and develop innovative approaches or workflows to solve problems in work (Janssen and Van Yperen, 2004; Hirst et al., 2009; Anderson et al., 2014). Performance-oriented individuals evaluate their own capabilities with job performance and ensure that their strengths are praised, or that their self-capabilities will not be denied or negatively evaluated through the impact of external environmental factors such as competition between work partners and acceptance of rewards given by the work team. VandeWalle (1997) divides performance goal orientations into two dimensions: performance-prove and performance-avoid. The performance-prove orientation reveals that individuals keep looking for good judgment that demonstrates their strengths and excellent work capabilities through external rewards, authorization or sharing of decisions. The performance-avoid orientation identifies more with entity theory than the performance-prove orientation (VandeWalle, 1997). The performance-avoid orientation believes that the capabilities of an individual remain unchanged, and that success is subject to the connate ability. Individuals of this orientation worry that managers or work partners give negative evaluations of their job performance and capabilities, and these performance-avoid individuals tend to retreat from failures and setbacks and thus choose goals which are more easily achieved (Dweck, 1986). Thus, they lack interest in difficult tasks (VandeWalle, 1997).

The model of goal-oriented behavior suggests that “intentions” are a key antecedent to anticipate individual behaviors (Soscia et al., 2018; Ajzen, 2020). According to views of the SOR model, individual behaviors and performance can be regarded as the same outcome, and the differences in subsequent behaviors and performance response will depend on the psychological cognition and attitude of individuals. In particular, intention can also be regarded as psychological or attitudinal trait, which highlights the transformation of individuals’ internal mechanism (Yang et al., 2021; Zhang et al., 2021). Many scholars studying behaviors hold the same idea and demonstrate in empirical studies that there is a very stable relationship between the two dimensions. To cite an example, Ajzen (1991) finds that intention is an important variable that drives behavioral expressions (Sahu et al., 2020). Similarly, La Barbera and Ajzen (2020) verify this relationship in their integrated analytical research. Thus, the discussion of teachers’ teaching innovation performance is not limited to how to change teaching contents, but also checks whether teachers have a high degree of teaching innovation intention and involves further understanding of changes in intention and performance through the view of intrinsic motivation. Based on the above statements, we contend that a higher innovation intention of teachers in teaching will lead to a higher possibility of having innovation performance in teaching. Thus, the study develops a hypothesis as follows:

H1: Teachers’ teaching innovation intention has a positive effect on teaching innovation performance.

### Innovation Attitude

Attitude has been a topic of wide concern among psychologists, but it has distinct definitions and natures in different academic fields and empirical situations (Soscia et al., 2018). In the research field of individual behaviors, marketing scholars widely accept the arguments of Ajzen (2020). The two psychologists suggest that attitudes (i.e., for smoking or innovation behaviors) should be defined based on behaviors in specific situations. It is a widely held view among scholars that attitudes form when individuals express their psychological tendency (degree of like and dislike) toward specific matters or behaviors through the reflection and evaluation process (Greenwald and Banaji, 1995). Other researchers regard attitude as a learned behavioral tendency, believing that attitudes take shape in the learning process, and grow to be stable and strengthened through repeat learning (Ajzen et al., 2018). This is why decision makers autonomously start the evaluation process under a particular situation (e.g., with specific actions or matters). This is a process of memorizing and associating specific matters or behaviors (Greenwald and Banaji, 1995; Ajzen et al., 2018). Thus, attitudes are generally recognized as passive reactions. Ajzen (1991) expresses the same view in his theory of planned behavior, and confirms that attitudes can affect individual behaviors through the mediating role of intentions. A great number of subsequent empirical studies also verify that attitude mentioned in the theory of planned behavior is an important factor influencing behavioral intentions (La Barbera and Ajzen, 2020). Based on the above statements, we develop a hypothesis as follows:

H2: Innovation attitude in teaching has a positive effect on innovation intentions in teaching.

### Positive Anticipated Emotions

Prior studies of behavioral decisions verify the theory of planned behavior (Ajzen, 1991; Sahu et al., 2020) as a model that can effectively explain consumer behaviors. However, for the explanatory power for behaviors in different situations, this theory still has some room for improvement. Thus, two consumer psychologists further propose the model of goal-oriented theory based on modifications to the theory of planned behavior (Perugini and Bagozzi, 2001). In the model of goal-oriented theory, Perugini and Bagozzi (2001) include a significant psychological concept: anticipated emotions. Scholars in this field argue that anticipated emotions exert an influence on behaviors mainly through the future-oriented behavioral goals and forward-looking thinking of humans. In other words, in addition to the existing experience (i.e., acquired attitude), individuals will take into account the goal achievement process under future circumstances while making decisions. Specifically speaking, anticipated emotions refer to decision makers’ anticipated emotional responses to success and failure based on forward-looking thinking in the current time frame. The formation of anticipated emotions requires the process of forward-looking counterfactual thinking (Legros and Cislaghi, 2020) that happens at the time when individuals make a decision. This process is also known as prefectural thinking (Epstude and Roese, 2008). For example, when an individual sets a goal and evaluates the possibility of success based on previous facts, he/she will have positive emotions such as joy if the goal is estimated to be easily achieved. These positive emotions can be deemed the drivers for individual behavioral decisions, and will further affect actions through intentions, thus facilitating and guaranteeing the goal achievement.

Attitude and anticipated emotions are two key concepts embedded in psychology, but in essence they vary, to some extent, from each other (Perugini and Bagozzi, 2001). First of all, they are different in referents. Attitude focuses on what an individual does, so its referent is the action itself, while anticipated emotions concentrate on whether an individual can achieve his/her behavioral goals, but not on actions (Soscia et al., 2018). Second, they have different processes of formation. Attitude takes shape in the process of learning, based on which the tendency toward a particular thing or action forms, so attitude is relatively stable within a short period of time. However, anticipated emotions take shape in a more dynamic process, which may be adjusted based on the degree of achievement of goals (Pintrich, 2003). In other words, an individual sets behavioral goals first and then anticipates the possibility of achievement through the forward-looking evaluation process, thus generating positive or negative emotions. Third, attitude and anticipated emotions are measured in different ways. Attitude is measured by the bipolar semantic differential items, and allows decision makers to choose the degree of like and dislike (Ajzen, 2020). Teachers’ teaching innovation intention is a pattern of attitude, and the change and intensification of attitude will depend on the impact of psychological motivation and factors. However, few studies discuss how teachers’ anticipated emotions affect the teaching innovation intention. Thus, this study aims to further discuss the intrinsic impact of anticipated emotions on teaching innovation intention.

The above descriptions of differences illustrate that anticipated emotions are not passive reactions based on previous experience, but take shape on the basis of decision makers’ anticipation of future actions and of process feedback (Guimond et al., 2014). Decision makers will make a detailed plan for the process of achieving goals through imagining the goals will be achieved. At the same time, decision makers make every effort to avoid the possibility of future disappointment (caused by failure in achieving goals), and they create scenarios that are conducive to the generation of positive emotions. For example, as indicated by Bagozzi and Pieters (1998), positive anticipated emotions have a significant impact on behavioral intentions, thus leading to goal-oriented behaviors. In addition, Guo et al. (2020) find that positive anticipated emotions offer the basis of motivations and then affect actions and drive decision makers to invest resources for positive expected outcomes. Based on the above statements, this study develops a hypothesis as follows:

H3: Positive anticipated emotions have a positive effect on innovation intentions in teaching.

### Group Norms

Group norms refer to the common codes of conduct, attitudes or values, as perceived by individuals, that are supposed to be followed in a group (He et al., 2012). When an individual perceives that other teachers in a group set a task (e.g., service innovation) as an important goal, he/she will also tend to regulate himself/herself with such a goal or internalize it as his/her code of conduct (Kelman, 2006; Guimond et al., 2014). The theory of social influence reveals that group norms guide teachers’ behavioral decisions through the process of internalization (Kelman, 2006; Chaker and Impedovo, 2021). In the context of teaching innovation, although a teacher can decide the degree of teaching innovation based on personal considerations, he/she will feel stressed when his/her services provided deviate from the common goal of the group (He et al., 2012; Soscia et al., 2018). As time goes on and with the accumulation of experience, the teacher will integrate the group norms into his/her own action objectives (Thomas et al., 2009). Thus, when he/she provides services, the teaching innovation will also be considered as an important task in the service process. To put it differently, he/she will take into account the expected goals of the group as they relate to services, thus forming a norm-based pressure (Kelman, 2006; Thomas et al., 2009). The teacher who highly perceives the group norm will keep investing resources to achieve the goal pursued by the group, which further facilitates the innovation intentions. On the contrary, a low level of group norms signifies that the group lacks an identity with task goals and motivations for efforts (Høigaard et al., 2006), resulting in a weak group consensus. Given this, we contend that the intention of achieving innovation goals will enhance with the increasing perception of the group norms for innovation. Based on the above statement, we propose a hypothesis as follows:

H4: Group norms have a positive effect on innovation intentions.

### Social Identity

Social identity means that a teacher perceives that he/she has common grounds with other colleagues and considers himself/herself as a part of the team or school (Fujita et al., 2018). Generally speaking, social identity is an integrated part of an individual’s self-concept and his/her bond to the society, school or group that he/she belongs to, including the value of relationship and emotional bond (Tajfel and Turner, 2004; Kim et al., 2018). An individual can define himself/herself through this process, and relevant elements for construction of a self-concept in this process include social category, social status or social state. Individuals can significantly perceive that they are a part of their group and different from individuals outside the group when the social identity is taking shape (Hogg and Terry, 2014). The social identity derives from the influence of the social group that an individual belongs to, but sometimes individuals also identify with some social groups with which they never interact. Take a particular environmental group as an example. Although individuals never have direct interaction with this environmental group, they share cognitive values, which drives individuals to consider themselves as a member of the group.

In essence, social identity is composed of three elements (Chiu et al., 2006; Meyer et al., 2006): cognitive social identity, affective social identity and evaluative social identity. The cognitive social identity signifies that a teacher perceives his/her identity as a teacher in the social group through the self-categorization process. This process highlights the common grounds between individuals and the group, which makes teachers spontaneously differentiate themselves from other non-teacher individuals. The depersonalization will appear when the degree of social identity is extremely high (Hogg and Terry, 2014). The affective social identity means the affective commitment to a group. A higher commitment level will lead to affective attachment (Chiu et al., 2006). With regards to the evaluative social identity, the positive value connotation, that is, organization-based self-esteem, of teachers in the group originates from the self-value evaluation of all teachers in the group (Bergami and Bagozzi, 2000). With the three elements of social identity, teachers will be more loyal to a school when they contribute more to the school (Chiu et al., 2006).

Social identity theory (Tajfel and Turner, 2004; Kim et al., 2018) reports that a teacher will express his/or social identity with specific actions to demonstrate his/her identity if he/she classifies himself/herself into the in-group teacher. From a different perspective, Rather (2018) argues that a stronger identity with the group will guide a teacher to spontaneously seek benefits for the group, and even build an awareness of a community with a shared future. Alden and Cheung (2000) also demonstrate that teachers with a higher sense of identity will carry out activities that facilitate the positive growth of the group, and even have proactive citizenship behaviors. All these studies share the idea that teachers with a higher sense of identity tend to have a high sense of social responsibility and actively contribute to the improvement of teaching content. In other words, teachers will recognize the responsibilities and obligations of their education, set higher requirements for students’ learning outcomes, and be more willing to make contributions to teaching innovation when they have a high degree of social identity. Thus, this study infers that teachers with a higher sense of identity will have higher innovation intentions. Based on the above discussion, the study develops a hypothesis as follows:

H5: Social identity has a positive effect on innovation intentions.

**FIGURE 1:**
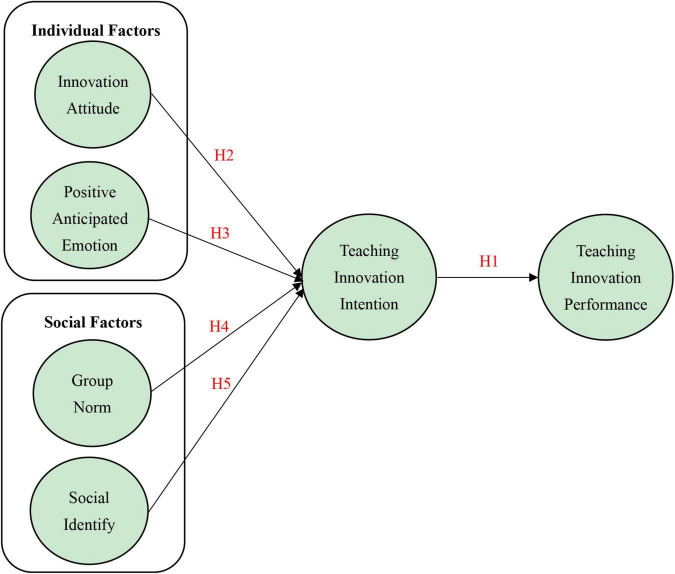
Research framework.

According to the above hypotheses, the research framework is shown in Figure 1.

## Methodology

### Sampling

This study aims to understand the impact of knowledge governance on teachers’ knowledge transfer and sharing. As there are different quarantine procedures in different countries, and the pandemic has different influences on people’s psychological characteristics, it is impracticable to take each country as a sample. In order to verify the role of the western theoretical model of social identity in high schools in Asia, this study collects samples from the Chinese mainland. Thus, we adopt purposive sampling and will establish several conditions during sampling so as to improve the representativeness of the research samples. First, we selected mainland China, where the pandemic was most severe at its beginning, as the main area for sampling, and its quarantine policy was the strictest. Thus, the sample is representative to a certain extent. Second, to understand the status of teaching innovation, it is necessary to focus on teachers, and the study adopted high schools as the main sampling context. This study takes the teachers at high schools, excluding the staff in the administrative department, as the study population in order to accurately collect representative samples. The questionnaire informed participants of the research purpose, research ethics and low risks, and the questionnaire information was processed in an anonymous way. In this study, we sent copies of the electronic questionnaire and collected 483 questionnaire copies. After excluding eight invalid copies, 475 valid questionnaires remained. Regarding the demographic information of participants, 73.2% of teachers were male and 26.8% female. Most teachers gave more than 30 h of lectures a week (85.5%) and a majority of these teachers were 30–35 years old (63%) and had an average length of teaching of 7.3 years.

Furthermore, the study collects information from the same respondents in form of a single questionnaire, which may lead to the common method bias (CMB). In this study, the single factor verification from Harman is adopted and all the measured items are analyzed by the non-rotating matrix. The analysis results demonstrate that there are nine factors, of which the eigenvalue is greater than 1, and the explanatory variance of factor 1 is 31.63% that could not explain most of the variance. Therefore, it can be concluded from the verification results that there is no common method bias in this study.

As there may be a reaction bias between the recalled and unrecalled samples, the recalled questionnaires were divided into the early group and the late group, as suggested by Armstrong and Overton (1977). A verification was conducted to check whether there were significant differences between the two groups in terms of the sample data and the study dimension. The verification results show that there is no significant difference in terms of the main dimensions and the basic information, which indicates that the problem of non-response bias is not significant.

### Measures

In the construct measurement part of this study, the Back Translation method is used, which is a scale developed by scholars in the past. The researcher invites an expert who is proficient in both Chinese and English to translate the original English items into Chinese, and invites another bilingual expert to translate the Chinese into English without contacting the original scale. A scholar in the field of marketing conducts final review to ensure that the meaning expressed by the questions is consistent with the original scale (i.e., the original English questionnaire and the translated English questionnaire).

For the measurement of teachers’ teaching innovation performance, this study refers to the scales of previous studies (Anderson et al., 2014; Al-Azmi, 2018), asking employees to self-evaluate their innovation performance. The measure of teachers’ teaching innovation intention is mainly compiled with reference to the research of Ajzen (1991), Chaker and Impedovo (2021), which consists of two measurement items. The measurement of innovative attitude is mainly compiled with reference to the scale of Ajzen (1991), which consists of two measurement items. The measure of Positive anticipated emotion is mainly compiled with reference to the research of Bagozzi and Pieters (1998). It mainly evaluates the emotional response of employees to achieve the service innovation goals set by them in the future. Group norm is measured by the items of Bagozzi and Lee (2002), which mainly evaluate the normative pressure of group innovation felt by designers. Social identify is measured by the item developed by Meyer et al. (2006) and mainly evaluates teachers’ sense of identity with the school.

### Analytical Tool

This study adopted structural equation modeling to test research hypotheses. In order to confirm reliability and validity, confirmatory factor analysis (CFA) was performed using SmartPLS 3.0 and SPSS 25.0. Compared with CB-SEM, PLS-SEM is more suitable for this study including when the research objective is exploratory research for theory development; when the analysis is for a prediction perspective; when the structural model is complex; when the structural model includes one or more formative constructs; when the sample size is smaller due to a small population; and when distribution is lack of normality. The above reasons provide supports to consider the PLS is an appropriate SEM method for a study. Finally, partial least squares structural equation modeling (PLS-SEM) was conducted to verify the structural model *via* SmartPLS 3.0.

## Data Analysis

### Assessment of Measurement Model

SmartPLS 3.0 and SPSS 25.0 were used to analyze the data. Before testing the hypotheses, the validity of the instrument was evaluated using convergent validity and discriminant validity. In addition, confirmatory factor analysis (CFA) was used to evaluate the measurement model. The data test results showed that the Cronbach’s α values of all the constructs were above 0.857 in Table 1. According to the study results, the reliability was significant when the Cronbach’s α coefficient exceeded 0.7, which indicated that the internal consistency of each construct was high. The AVEs and CRs of all dimensions in the Table 1 are all higher than the recommended value of 0.5 and 0.8, so all the dimensions of this study has good convergence validity. In addition, divergent validity was tested by comparing the average variance extracted (AVE) for each construct with the square of correlation coefficients. AVE for each construct was greater than the square of the related correlation coefficients, indicating the divergent validity of the constructs.

**TABLE 1:** Scale measurement.

	1	2	3	4	5	6
1. Innovation attitude	*0.859*					
2. Positive Anticipated Emotion	0.285**	*0.874*				
3. Group Norm	0.203**	0.518**	*0.897*			
4. Social Identify	0.408**	0.528**	0.405**	*0.884*		
5. Teaching innovation intention	0.586**	0.415**	0.322**	0.374**	0.867	
6. Teaching innovation performance	0.354**	0.751**	0.500**	0.547**	0.447**	*0.891*
Cronbach’s α	0.833	0.900	0.885	0.842	0.844	0.893
AVE	0.737	0.764	0.804	0.782	0.752	0.794
CR	0.917	0.942	0.944	0.891	0.924	0.932

**p < 0.01.

### Hypothesis Testing

Before the analysis, this study first analyzed the fit of the structural model (Geisser, 1974). Then, Stone-Geisser-Criterion (Q^2^), coefficient of determination (R^2^), and standardized root mean square residuals (SRMR) is used to assess the overall model fit. In our results, Q^2^ values were above 0, all R^2^ values were more significant than 0.10, and SRMR was less than 0.08, meeting the expected criteria (Götz et al., 2010). Variance inflation factors (VIF) in all paths will be checked to determine whether there is an issue with multicollinearity in this model. In all the paths, the largest VIF value is 1.872, so there is no such a problem.

**FIGURE 2:**
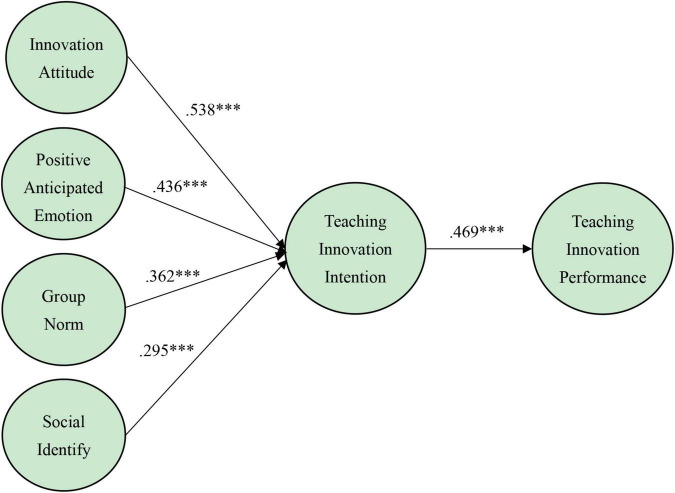
Structural model. ^***^p < 0.001.

Figure 2 provides the results of testing the hypotheses. This study also tested for direct effects between the variables and derived the degree of effect. Regarding H1 and H2, the results indicate the positive and significant effects of innovation attitude (β = 0.538, *p* < 0.001) and positive anticipated emotion (β = 0.436, *p* < 0.001) on teachers’ teaching innovation intention. So H1 and H2 are supported. Moreover, the results show that group norm (β = 0.362, *p* < 0.001) and social identify (β = 0.295, *p* < 0.001) have positive and significant effect on teachers’ teaching innovation intention, which supporting H3 and H4. Similarly, teaching innovation intention (β = 0.469, *p* < 0.001) has positive impact on teachers’ teaching innovation performance, so H5 is confirmed.

## Conclusion

### Discussion

In the face of diversified teaching environments, teaching innovation has become a key method for schools to create learning value for students. Many scholars also propose that teaching innovation is an important basis for students’ learning engagement (Al-Azmi, 2018); therefore, how to maintain and promote teachers’ innovative teaching behavior is an important topic for scholars and school principals in the field of education management. This study deepens the research of scholars in education service science and behavioral decision-making. This study discusses teachers’ teaching innovation intention from the individual and social perspectives to provide a more improved research framework and great insights into education innovation.

This study assumes that teachers’ teaching innovation intention will have a positive effect on teaching innovation performance. The results show that teachers’ teaching innovation intention has a positive and significant effect on teaching innovation performance. This agrees with the findings of Ajzen (2020), Soscia et al. (2018), Sahu et al. (2020) that teachers with much willingness to innovate in the contents of teaching courses will have a more significant innovation performance. This also signifies that teachers’ attitude toward innovation is an antecedent indicator that will affect their subsequent innovation behaviors, and that it would be impossible to guide teachers to present the substantial teaching innovation performance and outcome if there is no clear attitude or active intention.

Furthermore, this study examines the effect of intrinsic factors on teachers’ teaching innovation intention from the individual perspective. Results show that teachers’ innovative attitude and positive anticipated emotion have positive and significant effects on teaching innovation intention. This study deepens past research in the field of teaching innovation (Yuan and Woodman, 2010; Hadjilouca et al., 2011; Al-Azmi, 2018) and finds that positive anticipation emotion is an important factor affecting employee innovation performance. In the past, many studies based on the theory of planned behavior have mostly argued that internal attitude is an important factor affecting individual decision-making. However, many scholars (He et al., 2012; Soscia et al., 2018) believe that behavioral research should first consider the nature of behavioral decision-making in depth and then supplement it with appropriate theoretical foundations, so as to properly explain the decision-making process and its possible influencing mechanism. In essence, attitude is a psychological tendency formed based on past experience or situations; anticipatory emotion is a kind of “pre-psychological emotion” meaning that teachers will actively anticipate successful execution based on future service situations. This study is structured with the theory of goal-oriented behavior (He et al., 2012; Soscia et al., 2018) and integrated relevant literature on attitude and expected emotion (He et al., 2012; Chaker and Impedovo, 2021), further exploring the formation process of teachers’ teaching innovation performance. More importantly, this study finds that in the context of teaching innovation, positive anticipatory emotions are more likely to affect innovation performance than attitudes. Theoretically, this study finds and provides a new interpretation and point of view for service science and service innovation research, and can also be used as a reference for future research on teaching innovation behavior.

This study also discusses the effect of extrinsic factors on teachers’ teaching innovation intention from the social perspective. Results show that group norms and social identity have positive and significant effects on teaching innovation intention. This study invokes the theory of identity to further extend the past teaching innovation theory and explore the social-level influencing factors. In the past, research on teaching innovation behaviors mostly used group norms to explain teachers’ teaching innovation performance. This study finds that social identity can significantly enhance teaching innovation intentions; a recent meta-analysis by Khosravi and Chavan (2012) also supports this view. In practice, teachers with teaching innovation ability may not necessarily have positive innovation performance. Aiming to explain this situation, this study proposes a reasonable explanation: because the process of teaching innovation requires much effort, in addition to the ability to innovate, teachers need to have a considerable degree of organizational identity. This emotional connection will enhance teachers’ sense of responsibility for the school and their role, and they will then be willing to devote more energy to the process of teaching innovation. Even though the process of teaching innovation requires continuous trial and error and is full of challenges, the sense of identity can give teachers the motivation to overcome difficulties, persevere to the end (Tajfel and Turner, 2004; Fujita et al., 2018; Kim et al., 2018) and remain committed to improving students’ learning effectiveness and learning engagement. From a theoretical point of view, the investment in teaching innovation requires not only the input of resources at the cognitive level, but also the assistance of resources from group identification.

### Management Implications

The findings of this study have the following implications for service innovation practices; first of all, this study finds that expected emotion is a more effective mechanism for influencing service innovation than attitude. On the one hand, attitude is a process of learning and internalization that gradually forms a behavioral tendency; in essence, it is a more reactive influence mechanism based on accumulated information from the past. On the other hand, expected emotion is an emotional resource formed by an anticipation of a future situation, which is the driving basis for behavioral decision-making. In the past, many scholars also believed that emotion is an important resource in the innovation process, which not only motivates employees to conduct a broader creative search, but also increases teachers’ tolerance for innovation setbacks. This study suggests that principals or school administrators should design situations to stimulate teachers’ positive emotions in the process of teachers’ education and training, or invite teaching innovation models to speak out, so as to provide teachers with more teaching innovation ideas.

It can be seen from the positive effect of social factors on teachers’ teaching innovation intention that the establishment of smooth communication channels of internal information allows teachers to fully understand course contents and adjust the dynamic interaction with students, or further facilitate mutual support, thus forming a more unified atmosphere. Schools can provide sporadic training related to course teaching or collect student feedback through a teaching quality survey, so that teachers can perceive their school’s emphasis and efforts on teaching innovation. In addition, schools can establish incentives such as school-wide praise, annual awards and career promotion, or an internal incentive system to confirm teachers with good innovation performances and favorable innovations in teaching contents. In this way, teachers will make more contributions to shaping a favorable atmosphere of teaching innovation in schools when they perceive good teaching innovation performance models and criteria, and recognize schools’ emphasis on teaching innovation performance.

In general, appropriate innovation reward mechanisms send positive signals to teachers (i.e., information-level influences) and thus strengthen the link between behavioral intentions and innovation performance. In other words, when teachers recognize that the rewards of innovation performance give positive information, teachers will have inner confidence and a sense of competence, and then devote more efforts to the innovation process, so as to effectively strengthen the connection between innovation intention and innovation performance. Therefore, this study suggests that school administrators or principals can design incentive and reward systems related to teaching innovation, evaluate teachers’ teaching innovation models in a scientific way, provide teachers with more incentives to improve teaching conditions and give students more teaching innovation activities.

## Limitations

Although this study provides valuable insights into knowledge sharing and transfer among high school teachers, several limitations remain. First of all, this study discusses school governance from the perspective of knowledge governance. According to different theoretical perspectives, governance models will have more diversified governance models, including transactional governance, relational governance, etc. Therefore, it is suggested that future researchers can propose governance models that are more conducive to teachers’ knowledge sharing and transfer based on different theories, so as to increase the richness of governance theories.

Previous studies regard social capital as an important antecedent variable and discuss its effect on knowledge-sharing and -transfer behaviors. However, social capital can also be a moderator in the relationship between independent and dependent variables, and the social capital may be present in daily work, but not easily perceived by teachers. Therefore, future studies can discuss the moderating effect of social capital to offer more analyses and insights.

Furthermore, due to the limitation of time and funds, this study could not study high school teachers in different regions, but took high school teachers in the southeast coast of mainland China as a sample. Different regions may have large differences in teachers’ knowledge and school governance due to social and cultural differences. Therefore, this study suggests that future researchers can use regional factors as moderators to explore the influence of different regional factors on teachers’ knowledge-sharing behavior and whether there are different school governance models. Finally, due to the top-down management model of the school governance department, the leadership style of school principals may have an impact on their governance model, which may indirectly affect teachers’ knowledge-sharing behavior. Therefore, this study suggests that a multi-level model can be used to study the factors of a principal’s leadership style and team in the future, which is helpful to propose more meaningful theoretical contributions and practical significance.

## Data Availability Statement

The raw data supporting the conclusions of this article will be made available by the authors, without undue reservation.

## Ethics Statement

The studies involving human participants were reviewed and approved by Academic Committee of Minnan Science and Technology University. The patients/participants provided their written informed consent to participate in this study.

## Author Contributions

All authors listed have made a substantial, direct, and intellectual contribution to the work, and approved it for publication.

## Conflict of Interest

The authors declare that the research was conducted in the absence of any commercial or financial relationships that could be construed as a potential conflict of interest.

## Publisher’s Note

All claims expressed in this article are solely those of the authors and do not necessarily represent those of their affiliated organizations, or those of the publisher, the editors and the reviewers. Any product that may be evaluated in this article, or claim that may be made by its manufacturer, is not guaranteed or endorsed by the publisher.
